# 68Ga-PSMA-11 PET/CT Follow-Up of Patients with Prostate Cancer with Bone Metastases Who Had Reduced Bone Density after Androgen Deprivation Therapy

**DOI:** 10.3390/diagnostics11020277

**Published:** 2021-02-10

**Authors:** Mikhail Kesler, Ido Druckmann, Charles Levine, Jonathan Kuten, Ofer Yossepowitch, Einat Even-Sapir

**Affiliations:** 1 Department of Nuclear Medicine, Tel Aviv Sourasky Medical Center, 6 Weizmann St., Tel Aviv 6423906, Israel; mkezzler@gmail.com (M.K.); clevine789@hotmail.com (C.L.); jonathanku@tlvmc.gov.il (J.K.); 2 Department of Radiology—Musculoskeletal Imaging Unit, Imaging Division, Tel Aviv Sourasky Medical Center, Tel Aviv 6423906, Israel; idodru@gmail.com; 3 Department of Urology, Tel Aviv Sourasky Medical Center, Tel Aviv 6423906, Israel; ofery@tlvmc.gov.il; 4 Sackler School of Medicine, Tel Aviv University, Tel Aviv 6997801, Israel

**Keywords:** 68Ga-PSMA, PET/CT, prostate cancer, castration-resistance, ADT

## Abstract

Bone metastases from prostate cancer (PCa) often show an increase in density on computed tomography (CT) after successful androgen deprivation therapy (ADT). Density may be reduced, however, as the disease progresses or, contrarily, when disease is no longer active. The current study investigated the role of 68Ga-PSMA-11 positron emission tomography/computed tomography (PET/CT) in differentiating between these two conditions. Methods: The study cohort included 15 PCa patients with sclerotic/blastic bone metastasis in whom reduction in bone density of metastasis was noted on follow-up 68Ga-PSMA-11 PET/CT after ADT. Each patient had two PET/CT scans. Prior to the first scan, six patients were castration naïve and nine patients were already treated. All patients had ADT between the two PET/CT scans. PET parameters (SUVmax and tumor-to-background ratio), and CT parameters (HUmax) were determined and compared for each lesion on both scans. Patient’s response was based on prostate-specific antigen (PSA) levels and appearance of new lesions. The Kolmogorov–Smirnov test was used to evaluate normal distribution of the continuous variables. Results: Post-ADT reduction in bone density was identified in 37 lesions. The mean HUmax was 883.9 ± 175.1 on the first scan and 395.6 ± 157.1 on the second scan (*p* < 0.001). Twenty-one of the 37 lesions showed no increased tracer uptake on the second PET/CT scan raising the likelihood of a response. The other 16 lesions were associated with increased uptake suggestive of an active resistant disease. Bone density was not different in lesions that no longer showed an increased uptake as compared with those that did. Seven of the study patients responded to therapy, and none of the 16 lesions found in these patients showed increased 68Ga-PSMA-11 uptake. In eight patients with progressive disease, all 12 lesions in five of them showed increased 68Ga-PSMA-11 uptake, there was mixed response in two patients (having two lesions with increased uptake and one without) and although all three lesions no longer showed an increased uptake, new lesions were detected in the eighth patient. Conclusion: A decrease in density of bone lesions may reflect clinical progression, or contrarily, a response to therapy in patients with PCa and skeletal involvement treated with ADT. Uptake of 68Ga-PSMA-11 may separate between these two vastly opposing conditions.

## 1. Introduction

Prostate cancer (PCa) has a high propensity for metastasizing to the bone. As many as 75% of patients with advanced PCa have skeletal involvement. In autopsy, approximately 80% of men who died from PCa had bone metastases [1]. Prostate-specific membrane antigen (68Ga-PSMA-11) positron emission tomography/computed tomography (PET/CT) is highly sensitive and specific for detection of skeletal involvement in newly diagnosed prostate cancer patients. It has been shown to be superior to traditional bone scintigraphy (BS) as it allows for detection of lytic type and early marrow-based metastases in addition to osteoblastic-type metastases seen on BS [2,3,4]. In a recent study involving 112 prostate cancer patients, 68Ga-PSMA-11 PET/CT revealed bone metastases in 10% of the patients who were deemed to be non-metastatic on BS and in 36% of the patients with indeterminate BS [5]. 68Ga-PSMA-11 PET/CT is also valuable for identifying skeletal involvement in patients with biochemical failure after treatment [6,7,8].

The standard therapy for all patients with metastatic PCa is androgen deprivation therapy (ADT). While this treatment reduces bone pain and temporarily hinders metastatic growth, after a while the disease relapses and becomes castration-resistant prostate cancer (CRPC). Diagnosis of CRPC should be made as soon as possible in order to alter the treatment approach [9,10].

Monitoring the response of bone metastases to therapy has been an ongoing imaging challenge. PET tracers that accumulate in viable tumor tissue can separate between response to treatment showing a reduction in uptake that reflects a reduction in viable tumor cells mass and clinical progression that is characterized by enhanced uptake due to an increase in tumor load. Accumulating data suggest a benefit of sequential 18F-fluorodeoxyglucose (18F-FDG) PET/CT scans for assessing skeletal involvement in patients with 18F-FDG-avid disease [11,12,13]. In a similar fashion, we have found that a reduction in uptake of 68Ga-PSMA-11 in patients with metastatic prostate cancer reflects a response, while an increase in uptake and appearance of new lesions represent clinical progression [14].

In general, sclerosis of bone metastases on a follow-up CT after treatment indicates a response, while lysis, expansion of lesion, and detection of new lesions indicate clinical progression. In prostate cancer patients with skeletal involvement treated with ADT, a healing reaction is characterized by intensification of the osteoblastic response and sclerosis [11,12]. A reduction in bone density, however, is not necessarily due to progressive disease as ADT itself has a negative effect on bone mineral density with increased prevalence of osteoporosis [15,16].

We identified on follow-up 68Ga-PSMA-11 PET/CT scans performed after ADT treatment, bone metastasis that reduced in density on the CT data, both in patients that responded to treatment and those that did not, but with variable intensity of 68Ga-PSMA-11 uptake on the PET data. The objective of the current study was to identify the role of 68Ga-PSMA-11 PET/CT uptake for differentiating between response and active disease in bone metastases that show a decrease in density after ADT.

## 2. Materials and Methods

### 2.1. Patients

After receiving institutional review board approval, we searched our database to retrieve 532 patients with prostate cancer who had a 68Ga-PSMA-11 at our department between January 2019 and May 2020. In sixty-five patients with bone metastasis, a second PET/CT follow-up scan after ADT was performed during this time period. Of the latter, we identified 15 patients in whom ADT was associated with a reduction in bone density but with variable uptake of 68Ga-PSMA-11. These 15 patients comprised the study cohort. The mean patient age was 73.6 ± 6.7 (range 62–83 years). In six patients, the first PET/CT scan was part of cancer staging at presentation (hormone naïve) and in nine already treated metastatic patients, the first scan was performed during medical or surgical castration. All 15 patients received ADT before the second PET/CT scan. The distinction between patients who responded to ADT and those with clinical progression was based on changes in the prostate-specific antigen (PSA) level and the presence of newly diagnosed soft tissue and/or skeletal lesions on a follow-up imaging scan. In order to assess response and outcome, patients were followed for 7–22 months unless they died earlier. In nine of the study patients, a third PET/CT scan was performed, and its findings were also considered for assessment of response.

### 2.2. Positron Emission Tomography/Computed Tomography (PET/CT) Imaging

68Ga-PSMA-11 was injected intravenously as a bolus at a dose of 148–166.5 MBq between 50–100 min before acquisition was initiated. The patients were instructed to void immediately prior to acquisition. The PET/CT scans were performed from the tip of the skull to midthigh using Discovery 690 or MI PET/CT systems (GE Healthcare, Chicago, IL, USA).

Acquisition details for Discovery 690 PET/CT included the following: CT acquisition was performed using automatic mA-modulation and 120 kV. CT scans were reconstructed to a slice thickness of 2.5 mm. PET acquisition was performed with acquisition time of 3 min per bed position in three-dimensional (3D) mode. PET images were reconstructed in a matrix size of 128 × 128 with a pixel size of 5.5 mm and slice thickness of 3.3 mm. The reconstruction method was VUE Point FX by GE Healthcare that uses time-of-flight information and includes a fully 3D OSEM algorithm with 3 iterations, 24 subsets, and filter cutoff of 8 mm. The VUE Point FX algorithm also includes normalization and image corrections for attenuation, scatter, randoms, and dead time. A heavy Z-filter was applied to smooth between transaxial slices.

Acquisition details for Discovery MI PET/CT included the following: CT acquisition was performed using automatic mA-modulation and 120 kV. CT scans were reconstructed to a slice thickness of 2.5 mm. PET acquisition was performed with acquisition time of 3 min per bed position in 3D mode. PET images were reconstructed in a matrix size of 256 × 256 with a pixel size of 2.7 mm and slice thickness of 2.8 mm. Reconstruction method was VUE Point FX by GE Healthcare that uses time-of-flight information and includes a fully 3D OSEM algorithm with 3 iterations, 8 subsets, and filter cutoff of 6.0 mm. The VUE Point FX algorithm also includes normalization and image corrections for attenuation, scatter, randoms, and dead time. A heavy Z-filter was applied to smooth between transaxial slices.

### 2.3. Image Analysis

All scans were reviewed by nuclear medicine physicians (M.K., J.K., and E.E.S.), a body radiologist (C.L.), and a musculoskeletal radiologist (I.D.).

Any soft tissue or skeletal lesion showing above normal uptake and not associated with physiological uptake was considered to be a pathological lesion [17]. Typical pitfalls (i.e., benign and malignant lesions mimicking prostate cancer) in 68Ga-PSMA-11 ligand PET/CT imaging were considered (e.g., ganglia, fractures, sarcoidosis, etc.) [18].

Dense bone metastases identified on the first PET/CT scan which showed reduced bone density on the follow-up scan were recorded and analyzed, up to three lesions per patient. The maximum standardized uptake value (SUVmax) was measured using dedicated software (Xeleris™, version 4 DR, GE Healthcare, Milwaukee, WI, USA). The tumor-to-background ratio (TBR) was defined as the ratio of the lesions’ SUVmax and the SUVmax of healthy bone (background). Bone lesions with TBR higher than 1.7 were considered to be positive on PET. The maximum Hounsfield unit (HUmax) was determined for each bone site with the region of interest (ROI) measured inside the lesion. The pattern of density reduction in CT was categorized as either diffuse reduction or lytic lesion. The presence of new soft tissue and bone lesions was recorded.

A clinical response was defined as a combination of reduced uptake in all cancer lesions on a PSMA PET/CT scan and a decrease in prostate-specific antigen (PSA), without detection of a new positive sites on a second PSMA PET/CT scan. Clinical progression was defined as an increase of uptake in initial cancer lesions on PSMA PET/CT or new lesions detected by a second PSMA PET/CT with or without an increase in PSA.

### 2.4. Statistical Analysis

The Kolmogorov–Smirnov test was used to evaluate normal distribution of the continuous variables. Continuous variables were presented as mean and standard deviation (SD). A linear mixed model was used to compare HU and TBR between categories. All statistical tests were two sided and *p* < 0.05 was considered to be statistically significant. NCSS software was used for all statistical analyses (NCSS 2020 Statistical Software (2020), NCSS, LLC., Kaysville, UT, USA, ncss.com/software/ncss).

## 3. Results

Table 1 summarizes the PSA values, findings on PET/CT scans, and responses of patients to ADT therapy.

Thirty-seven metastatic dense bone lesions identified on the first 68Ga-PSMA-11 PET/CT scan showed a reduction in bone density on the second PET/CT scan performed after ADT. The mean HUmax on the first scan was 883.9 ± 175.1 and on the follow-up scan 395.6 ± 157.1 (*p* < 0.001). Of the 37 lesions, 21 were categorized as PET negative on the second PET/CT scan considered to be non-viable metastases and 16 were associated with increased uptake (PET positive), suggestive of active disease. The mean TBR ± SD for non-active lesions was 1.1 ± 0.3 and for lesions with active disease 16.3 ± 18.7, *p* = 0.003. Both groups of lesions did not differ, however, in bone density (mean HUmax ± SD of 376.9 ± 154.3 vs 420.3 ± 167.3, *p* = 0.419).

Five of the six hormone-naïve patients responded to ADT and all of their analyzed metastatic sites were PET negative concomitant to bone density reduction (Patients 1–5 in Table 1). In Patient 6 with progressive disease based on PSA levels and appearance of new metastases, three bone lesions with a reduction in bone density were categorized as PET positive.

Seven of the nine previously treated patients were diagnosed with clinical progression. In four patients, all nine lesions with a reduction in density were also PET positive (Patients 11 and 13–15 in Table 1). Two patients (Patients 10 and 12 in Table 1) showed a mixed PET response, since among three lesions that showed a reduction in density, two lesions were PET positive and one lesion was PET negative. In one patient (Patient 9 in Table 1), clinical progression was presented as the appearance of new lesions, however, the three previously seen metastases showed a reduction in density and were Pet negative.

Two of the nine previously treated patients responded to ADT as seen on the second PET/CT scan. In one patient (Patient 7 in Table 1), all three lesions that were PET positive on the first PET/CT scan became PET negative on the second scan; in another patient (Patient 8 in Table 1), three PET negative sclerotic lesions, seen on the first scan, remained PET negative on the second scan, but reduced in bone density.

Table 2 summarizes the response as assessed on the second PET/CT scan and clinical outcome of the patients based on follow-up including PSA levels and the findings on a third PET/CT scan. 

**Figure 1:**
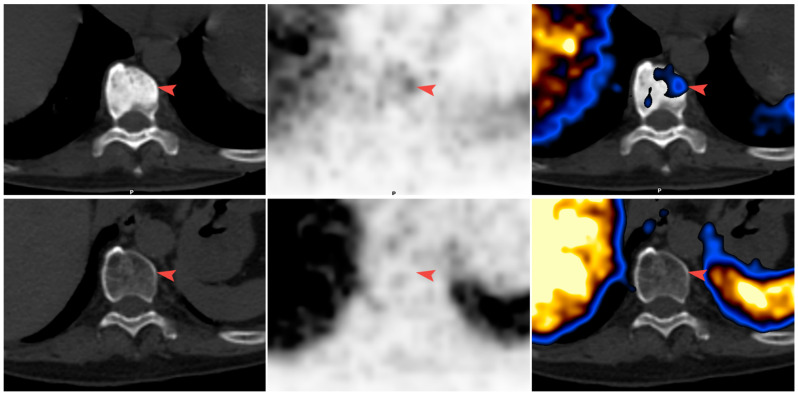
Disease response (Patient 7). (From left to right) CT; PET; and fusion images. In the first scan after ADT and bisphosphonate treatment, sclerotic metastasis in the vertebra was identified with very low uptake of 68Ga-PSMA-11 (arrowhead) (top row). In the second scan after ADT, the vertebra was no longer sclerotic and showed no uptake (bottom row). PSA levels were reduced and there was no evidence of new disease.

**Figure 2:**
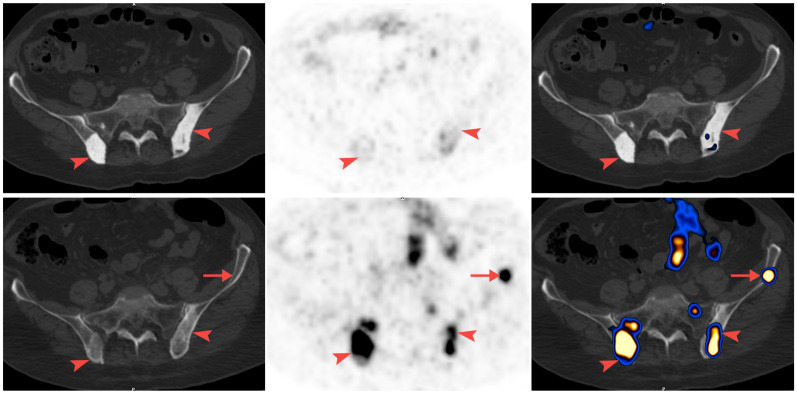
Clinical progression (Patient 14). (From left to right) CT; PET; and fusion images. In the first scan after ADT, sclerotic metastases in the vertebra were identified in pelvic bones with very low uptake of 68Ga-PSMA-11 (arrowhead) (top row). In the second scan, the lesions were no longer sclerotic and showed intense 68Ga-PSMA-11 uptake (bottom row). PSA levels increased and a new metastasis was detected (arrow).

Figure 1 and Figure 2 illustrate bone metastases showing a reduction in bone density. Figure 1 illustrates a response. The reduction in bone density seen on the CT is associated with a reduction in tracer uptake on PET, while Figure 2 illustrates bone metastasis showing a reduction in bone density and positive PET, in keeping with active disease.

## 4. Discussion

The current study addresses the challenge in differentiating between dense bone metastases of PCa that show a reduction in bone density because of active disease, probably representing castration resistance, and those that show a reduction as a response to ADT. We have found this phenomenon in approximately 25% of the prostate cancer patients with skeletal involvement treated with ADT in whom two PET/CT scans were available for comparison.

In healthy men, testosterone directly stimulates bone formation by inducing osteoblast proliferation. Testosterone, being a precursor to estrogen, also indirectly stimulates bone formation by inhibiting osteoclast function, hence, decreasing bone resorption. For decades, patients with a bone metastatic PCa have been treated with ADT. ADT induces a healing reaction that is characterized by intensification of the osteoblastic response and sclerosis; however, after some time, the disease relapses to CRPC. When resistance to antiandrogen therapy arises with low testosterone levels, bone metastases favor bone erosion more than bone formation; thus, a reduction in bone density may reflect resistance to ADT [15]. Ottewell et al. showed, in castrated mice, enhanced bone resorption and subsequent loss of bone density. By 2 weeks following castration, osteoclast numbers were increased [19]. 

Regardless of the metastatic disease, the hypogonadal state induced by ADT disrupts the normal bone physiology, having a negative effect on bone mineral density and increased prevalence of osteoporosis. Subsequently, men treated with ADT show an increase of 21–37% in fragility fractures [15,16,20]. Therefore, a reduction in bone density may be the result of opposite clinical scenarios, i.e., resistance to ADT and clinical progression or the contrary, successful ADT treatment, and reduction in density as a side effect of ADT. 

Monitoring the response of metastatic skeletal disease to treatment and early identification of castration resistance is an ongoing challenge. Measurement of PSA alone is not reliable enough for monitoring disease activity since visceral metastases may develop in men without rising PSA [4]. The Prostate Cancer Working Group (PCWG) 2 and 3 recommended a combination of bone scintigraphy and CT scans, PSA measurements, and clinical benefit in men with CRPC [21].

The development of bone sclerosis on CT in metastatic lesions has been suggested as reflecting a response in metastatic breast cancer within the MD Anderson Cancer Center criteria [22]. Sclerotic lesions usually remain unchanged during periods of remission [17,23]. However, there are no established CT density thresholds for distinguishing inactive bone metastases from active sclerotic metastases. Previous publications have suggested that a well-defined bone lesion with homogenous increased CT density (>800–1000 HU) is probably inactive [23,24]. This was not the case in the current study. Lesions with increased uptake suggestive of active disease and lesions that showed no increased uptake did not differ in bone density, and 14 of the active lesions that showed increased 68Ga-PSMA-11 uptake were associated with bone density of over 800 HU.

When a sclerotic lesion is altered to a lytic one on CT, the likelihood is in favor of active metastasis, as has been described in the progression of metastatic breast cancer [22]. It was also expressed in 1984 in a publication by Pollen et al. in a case series of four patients with blastic bone metastasis from PCa that became lytic on X-rays after ADT [25]. However, if no clear lysis is present but only the disappearance of sclerosis and a reduction in bone density, as was the case in the lesions analyzed in the current study, separation between responsive and progressive lesions cannot be obtained on CT alone. In contrast to CT and also to bone scintigraphy, PET tracers that accumulate directly in tumor tissue identify the presence of bone disease early in the process, when the tumor deposit is colonized in the marrow. The latter benefit of PET over BS and CT is also correct when monitoring response to therapy where a decrease in tracer uptake reflects a reduction in viable tumor tissue regardless of the change in bone morphologic appearance. This advantage was exploited by 18F-FDG PET/CT in various FDG-avid diseases as well as by labeled choline and, recently, by labeled PSMA in cases of PCa [26,27,28]. The findings of the current study suggest that uptake of 68Ga-PSMA-11 may differentiate between response and active disease. None of the 16 lesions identified in seven patients that responded to ADT, showed increased uptake. All the lesions found in five of eight patients with progression of disease were associated with high tracer uptake. Two additional patients had mixed response with some lesions showing uptake and one that did not, and an eighth patient’s lesions showed no increased uptake, but new lesions were detected. It appears that all patients with clinical progression had also PET/CT findings of either active bone metastases or new soft tissue lesions.

It should be noted that four of the hyperdense lesions that showed no increased uptake on the first PET/CT scan re-activated and showed increased tracer uptake along with reduction in density on the second scan, indicating that a lack of uptake in previously treated metastasis represents a response but does not necessarily mean a “burnt out” metastasis with total apoptosis.

There are several limitations to this study. The first is the small group of patients; however, the study included all available patients that fulfilled the selection criteria for the study.

The other limitation is the lack of histological validation in all lesions. As an alternative to histologic proof, the evaluation of the lesions was based on the clinical course for response characteristics, change in PSA, and the appearance of new lesions. 

## 5. Conclusions

In patients with PCa and skeletal involvement treated with ADT, a decrease in the density of lesions may reflect clinical progression or, contrarily, a response to therapy. Uptake of 68Ga-PSMA-11 may differentiate between these two conditions. Active disease is associated with increased tracer uptake, while there is no association with responsive lesions. The lack of increased uptake, however, should not be considered to be indicative of a non-viable metastasis.

## Figures and Tables

**Table 1:** PSA values, findings on PET/CT scans, and response to androgen deprivation therapy (ADT). Humax, maximum Hounsfield unit; TBR, tumor-to-background ratio; PSA, prostate-specific antigen; ST, soft tissue; AD, androgen deprivation therapy; T, Taxotere; BMA, bone-modifying agents; OE, orchiectomy; CP, clinical progression; R, response to therapy. * Treatment consisted of androgen deprivation therapy, Taxotere, bone-modifying agents, and orchiectomy. ** Patient response was either clinical progression or response to therapy.

Patient	Lesions	First Scan				Follow-Up Scan							Patient Response **
		Previous Treatment *	HUmax	TBR	PSA (ng/dL)	Treatment *	Months between Scans (mo)	New ST Lesions	New Bone Lesions	HUmax	TBR	PSA (ng/dL)	
**Castration-Naive**													
**1**	1		761	10	154.1	ADT	10	No	No	329	1.6	0.7	R
2	1		841	13.6	2114.8	ADT	12	No	No	137	0.5	0.1	R
	2		672	13						200	0.6		
3	1		822	2.5	17.4	ADT	5	No	No	496	1.4	0.1	R
	2		911	6.2						581	1.2		
	3		1099	7.2						418	0.6		
4	1		588	5	98.5	ADT	10	No	No	287	0.9	0.4	R
	2		616	5						384	1.0		
	3		827	4						589	0.9		
5	1		779	47.2	18.4	ADT	34	No	No	397	1.5	1.1	R
6	1		1187	3.1	169.7	ADT	28	Yes	Yes	585	37.6	350.0	CP
	2		1189	3.9						360	17.1		
	3		1105	5.5						463	10		
**Treated Before the First PET/CT**													
7	1	ADT + BMA	957	3.5	0.2	ADT	40	No	No	149	1.0	0.2	R
	2		837	3.5						171	0.9		
	3		963	2.6						194	1.1		
8	1	ADT + T	620	1.7	0.8	ADT + T	5	No	No	351	1.1	0.1	R
	2		651	1.7						329	1.1		
	3		605	1.5						401	0.9		
9	1	ADT + T	1028	5.9	0.2	ADT	22	No	Yes	384	1.5	0.6	CP
	2		1051	8.4						690	1.7		
	3		1070	5.3						576	0.9		
10	1	ADT	826	5.5	6.3	ADT	15	Yes	Yes	414	1.5	7.2	CP
	2		617	7.9						118	6.7		
	3		1181	6.2						188	4.2		
11	1	ADT + T	627	32.6	0.1	ADT + T	24	No	Yes	182	3.4	0.4	CP
12	1	OE	899	4.5	2.0	ADT	12	No	Yes	437	1.2	1.4	CP
	2		1014	5.0						453	7.4		
	3		880	3.3						341	22.1		
13	1	ADT	998	3.1	2.6	ADT	11	Yes	Yes	476	15.8	35	CP
	2		910	1						397	6.8		
	3		853	2.1						371	9.5		
14	1	ADT	1048	1.3	1.2	ADT	10	No	Yes	625	6.6	6.2	CP
	2		1030	1.2						698	6.7		
	3		902	2.6						595	19.5		
15	1	ADT + T	925	2	2.0	ADT	24	No	Yes	332	8.8	3.4	CP
	2		816	1.4						540	78.1		

**Table 2:** Clinical outcome on follow-up. pt, patients; m, months; CP, clinical progression; R, response to therapy; PSA, prostate-specific antigen. * In 9 patients a third PET/CT scan was performed and their findings were included in response assessment. ** Patients 9 and 14 were lost to detailed follow-up and the only data available was that there were alive.

Pt	Response According to PET/CT2	Follow Up after PET/CT2 (m)	Clinical Outcome on Follow-Up *
Castration-Naive			
1	R	7	Response (PSA)
2	R	9	Response (PSA and PET/CT3)
3	R	10	Response (PSA)
4	R	10	Response (PSA and PET/CT3)
5	R	22	Response (PSA and PET/CT3)
6	CP	3	Died (3 m after PET/CT2)
Treated before the first PET/CT			
7	R	15	Response (PSA and PET/CT3)
8	R	12	Response (PSA and PET/CT3)
9	CP	No FU	Alive for 7 m **
10	CP	4	Died (4 m after PET/CT2)
11	CP	17	Progression (PSA and PET/CT3)
12	CP	7	Progression (PSA and PET/CT3)
12	CP	11	Progression (PSA and PET/CT3)
14	CP	No FU	Alive for 15 m
15	CP	14	Response (PSA and PET/CT3) after adding chemotherapy

## Data Availability

Datasets used or analyzed during the current study are available from the corresponding author on reasonable request.
